# Determination of the Bending Properties of Wire Rope Used in Cable Barrier Systems

**DOI:** 10.3390/ma13173842

**Published:** 2020-08-31

**Authors:** Dawid Bruski

**Affiliations:** Department of Mechanics of Materials and Structures, Faculty of Civil and Environmental Engineering, Gdańsk University of Technology, Gabriela Narutowicza 11/12, 80-233 Gdańsk, Poland; dawid.bruski@pg.edu.pl; Tel.: +48-58-348-6149

**Keywords:** 3 × 7 wire rope, cable barrier, moment–curvature relationship, bending properties, modeling, numerical simulations, FEM, experimental tests

## Abstract

This paper presents research on the bending properties of 3 × 7 19-mm wire rope commonly used in road cable barriers. A total of 19 experimental tests were conducted. In addition, two nonlinear 3D numerical models of the wire rope using beam and solid finite elements were developed. Based on these models, four numerical simulations were carried out. The numerical results were validated against the experimental ones and a very good agreement was obtained. The main result of the research is the determination of the moment–curvature relationship for the wire rope considered. The effect of prestretching on the rope performance is discussed. The numerical results are analyzed in this paper in detail, including the behavior of the wire rope under bending and analyses of the cross-sectional and contact stresses. Suggestions concerning the type of finite element for wire rope modeling are also given. The results can be used, for example, in numerical simulations of crash tests of cable barriers.

## 1. Introduction

Wire ropes are in widespread use as structural members. A wire rope mostly consists of strands helically twisted around the central core. The strand is constituted of many wires helically wrapped around the inner wire. This implies that the rope geometry can be complicated [1]. Wire ropes are generally used to transmit tensile forces. They have high load-bearing capacities upon relatively small dead weights [2]. Moreover, the rope structure enables the wire ropes to resume loads despite the break of one or more wires [1]. Wire ropes are commonly used in civil and mechanical engineering, e.g., in bridges, cranes, cable cars, mine hoists, elevators, prestressed concrete structures, road safety equipment, and other similar applications [3,4,5,6,7].

Due to the increasing usages of ropes, numerous works investigating their performance have been published. They present, among other aspects, experimental tests [8], numerical tests [9,10], fatigue analysis [11], performances in various environments and conditions, and the behavior of partly damaged [1] or corroded wire ropes [5]. Developing a mathematical model for predicting wire rope responses is challenging due to the complex architecture of ropes, particularly if the effect of contact and frictional forces between wires is to be considered [12]. Most mathematical models are limited to a single, straight strand, where all wound wires are single helices [3,13,14]. However, most actual ropes consist of several or more strands in which most wires are configured as double helices [3,15]. To analyze ropes with a complex topology and to include various factors in calculations, the Finite Element Method (FEM) is increasingly being employed [16,17].

One analytical model was proposed by Costello (1997) [13]. It describes the equilibrium of a single wire, as well as static responses of a single strand and wire rope. Argatov (2011) [3] studied the response of a wire rope strand to axial and torsional loads. The refined discrete mathematical model was developed to correctly assess its deformation, taking into account interwire contacts. This model enables local contact stresses to be obtained. Argatov et al. (2011) [4] studied the wear degradation of wire ropes subjected to cyclic bending over a sheave by applying the Archard–Kragelsky wear law. The proposed mathematical model can be useful for estimating the fatigue life of wire ropes.

**Figure 1:**
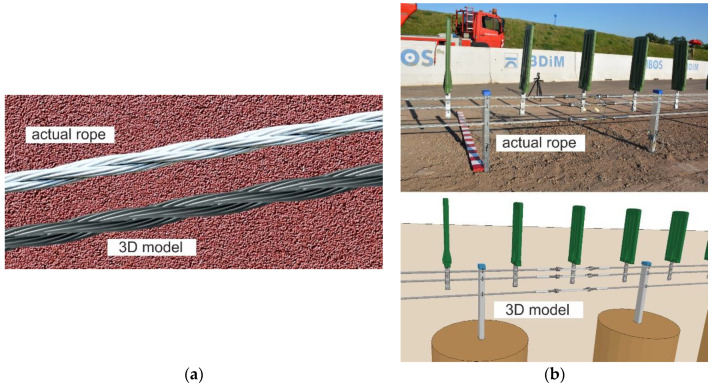
Wire rope models: (**a**) Detailed 3D model (for short section); (**b**) simplified (for long rope section, here used in a cable barrier model).

Nowadays, numerous research employing numerical simulations is being conducted. FEM simulations make it possible to analyze the response of wire ropes in detail, even for a complicated geometry. The most valuable simulations are those compared and validated against experiments. Generally, numerical models of wire ropes can be divided into 2D (simple) [2] and 3D (more complex) formulations [12,18]. 3D models are more common and universal. These models are usually developed using solid finite elements (FEs) or beam FEs [2]. The simulations, where solid FEs are applied, are high-cost computational methods [2]. There are also so-called mixed models utilizing solid and beam FEs at the same time. For instance, the core can be modeled as solid FEs and the wires as beam FEs [19]. Advanced and detailed 3D models (Figure 1a) are typically used to analyze short sections of ropes because they require much work and computational resources. For analyses of a rope of a considerable length (e.g., several dozen meters, Figure 1b), simplified models are commonly employed [11,20].

Previously, numerical analyses were limited to simple straight strands; currently, they are used to examine geometrically complex ropes. Jiang et al. (2000) [16] developed a numerical model of a wire rope utilizing solid FEs. The elaborated model showed better agreement with the experimental results from the literature than the analytical model proposed by Costello in 1997. Elata et al. (2004) [15] developed a new model for simulating the mechanical response of a wire rope subjected to both an axial load and an axial torque. The model was validated against experimental data collected from the testing of two cables and compared to Velinski’s and Costello’s model. Erdönmez et al. (2009) [21] numerically studied the axial loading and bending of simple strand wire over a sheave. The results showed that the maximum stress occurred over the upper sheave midpoint. Judge et al. (2011) [22] conducted experimental and numerical research on a cable consisting of 120 wires subjected to the impact of a 20-mm fragment with velocities between 200 and 1400 m/s. The numerical simulation showed good agreement with the laboratory tests. Boroška et al. (2014) [23] described the influence of various factors on Young’s modulus of the wire ropes and gave some values. They also numerically investigated the 100-mm long sample and presented the stress distributions within the rope. Wu (2014) [10] developed a numerical model of a wire rope subjected to tensile loads and compared the results to theoretical and experimental data taken from the literature. Foti et al. (2016) [24] investigated the behavior of a strand under axial–torsional loads using a new analytical formulation and FEM simulations. The results were validated against experimental and theoretical results from the literature. The effects of wire lay angles and the torsional boundary were discussed. Karathanasopoulos et al. (2017) [2] proposed a 2D elastoplastic FE model to simulate the behavior of a simple strand. The advantage of this simplified planar model is its robustness and low computational cost. The results were compared with the 3D FE model of Foti et al. [24] and a very good agreement was obtained. Vukelic et al. (2017) [12] numerically investigated the influence of a reduction of the cross-sectional area on stress levels and the remaining fatigue life for three design types of wire ropes. The results showed that the reductions in cross-sectional areas below 90% of the initial area cause a substantial increase in stress levels and significant decreases in the remaining life of the wire ropes. Wang et al. (2017) [17] developed an FE model of wire rope and showed that stresses are unevenly distributed within the rope. They also conducted experimental tensile tests and compared the results with the simulation. The calculations showed good agreement with physical tests. Smyth (2018) [11] analyzed a wire rope used in an overhead crane and, because the actual bending stiffness properties were not known, the author applied data from another wire rope type and appropriately scaled it using the relative stiffness ratios between the ropes. The papers cited above indicate that both theoretical and numerical models still need to be developed, tested, and then refined. It is noteworthy that publications dealing with the bending properties of wire ropes are still limited.

**Figure 2:**
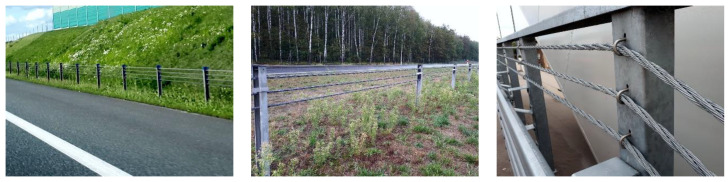
Examples of safety barriers using 3 × 7 19-mm wire rope.

In this paper, the subject of the analysis is a 3 × 7 19-mm wire rope, which is commonly used in road cable barrier systems (Figure 2) [25,26,27]. Descriptions of the cable barrier systems and examples of their use are presented in previous reports [28,29,30,31,32]. Knowledge about this wire rope and its properties is crucial, helping to better understand and analyze vehicular impacts on cable barriers. So far, research has mainly focused on evaluations of the performance of the whole safety system [33,34]. To the best of the author’s knowledge, the only studies on the properties of the 3 × 7 19-mm wire rope have been published by the team led by Reid [20,35,36]. To determine the bending properties, they tested the wire rope in a cantilever position. The longest tested rope was 1.359 m long, and the maximum applied point load was approximately 36.5 N. The physical tests were supplemented with numerical simulations. The rope was modeled in a simplified manner, using only one beam FE in the wire rope cross-section, because these studies were concentrated on the development of a wire rope computational model which can be used in applications of a considerable length. It should be mentioned that in the literature, there are no studies on four-point bending of this rope. Due to the small amount of experimental data and the lack of numerical analyses using advanced 3D models, further research on 3 × 7 19-mm wire rope is desirable.

This work aims to determine the bending properties of the 3 × 7 19-mm wire rope. The main result of the research is the determination of the moment–curvature relationship. The other objectives are to analyze the wire rope behavior under a bending condition, to investigate the interwire contact and to test whether the prestretching affects the rope bending performance. The practical aspects of the article are to determine and explicitly deliver equations for recreating the actual 3D geometry of the rope, to present the methodology of developing advanced numerical rope models, and to give suggestions concerning the use of beam or solid FEs. To achieve the assumed objectives, 19 experimental tests were carried out, which were supported by four nonlinear FEM analyses. With reference to published research on the 3 × 7 19-mm wire rope, this study presents the results of four-point bending tests and the effect of prestretching on the rope bending properties and delivers the equations enabling one to recreate the 3D 3 × 7 wire rope geometry. Furthermore, conducted numerical simulations will supplement knowledge on the contact stress values and their distribution within the rope, as well as the influence of the friction coefficient between the wires on the rope performance. The previous models of this rope, collected and published in [36], do not allow for the analysis of the mentioned issues, as they were developed for a different purpose. This research is important because the wire rope analyzed is used in road cable barriers and its properties affect the effectiveness of the whole safety system, thus affect the safety of vehicle occupants during an accident. Taking into account the small amount of available experimental and numerical data, it is advisable to conduct further studies allowing for a better understanding of the wire rope behavior. This data could potentially give suggestions for the optimization of the 3 × 7 wire rope, for instance, in terms of the dimensions and geometry. The study will also give suggestions for conducting experimental and numerical bending tests for wire ropes. The remainder of this paper is organized as follows: Section 2 describes the characteristics and the geometry of the wire rope; Section 3 presents the moment–curvature relationship from the Bernoulli–Euler beam theory; Section 4 deals with the four-point experimental bending tests, introducing the test stand and specimens and presenting the results; Section 5 shows the computational models developed and an analysis of numerical simulations; in Section 6, the final moment–curvature relationship is proposed and the research is discussed; and Section 7 contains the summary and conclusions.

## 2. The 3 × 7 19-mm Wire Rope

### 2.1. Characteristics of the Wire Rope

**Figure 3:**
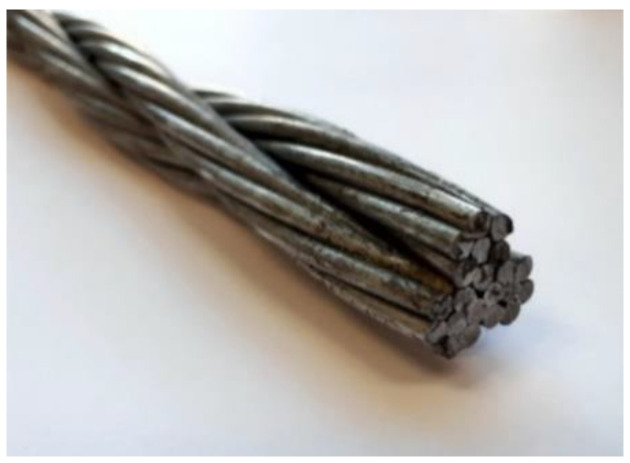
3 × 7 19-mm wire rope used in road cable barriers.

The subject of this study is a steel 3 × 7 wire rope (Figure 3), which is commonly used in road cable barriers. The wire rope consists of three strands and each strand consists of seven 3 mm diameter steel wires; thus, the whole wire rope consists of 21 wires in total. The nominal rope diameter is 19 mm (0.75 in.). The wire rope geometry is described in Section 2.2.

The author’s laboratory tests have shown that the rupture load of one wire is approximately 10 kN and for one strand (i.e., seven wires), is approximately 70 kN. Based on these values, it can be concluded that the breaking load of the wire rope should be approximately 210 kN. This value can be confirmed in the literature, e.g., the publications [20,35,37] show that the load-carrying capacity of the 3 × 7 19-mm wire rope obtained from quasi-static tensile tests is approximately 200 kN. The Young’s modulus of the non-prestretched and prestretched wire rope is 79.9 and 116.1 GPa, respectively [35]. The author’s experimental tests confirmed these values. Moreover, the report [29] states that an effective modulus of non-prestretched rope is approximately 76–90 MPa and that the effective modulus of the wire rope in safety barriers can increase by almost 50% after two years of installation due to cyclic loading from temperature variations and proper field monitoring of the system. The cross-sectional area of the wire rope is 148.4 mm^2^. The material density is equal to 7948 kg/m^3^ [35].

Observations from real road accidents have revealed that cable barriers, whose main longitudinal structural elements are wire ropes, are capable of containing vehicles exceeding design loads of the cable barrier system [29] and rope fracture rarely occurs in cable barrier systems [35]. The values of the forces in wire ropes during a road accident mainly depend on the speed, impact angle, mass, and geometry of the vehicle. The maximum wire rope tensile load during a full-scale crash test, when a 2034-kg Chevrolet C2500 impacted a 3-cable barrier system at 98.1 km/h and 26.2°, was 108 kN [20,35]. The maximum load recorded during an experimental test of the perpendicular impact of a 772-kg boogie vehicle on a single wire rope at the speed of 24 km/h was 165 kN [20]. Numerical simulations of the impact of a 1500-kg passenger car on a 4-cable barrier at the speed of 110 km/h and at the angle of 20° revealed that the forces in the rope can reach 76 kN [38]. The striking of a Heavy Goods Vehicle (HGV) weighing 38 tons into a 3-cable barrier at the speed of 65 km/h and at the impact angle of 7° can exert forces on one wire rope of up to 50 kN [39]. These values are below the rupture load of the wire rope.

### 2.2. Geometry of the Wire Rope

**Figure 4:**
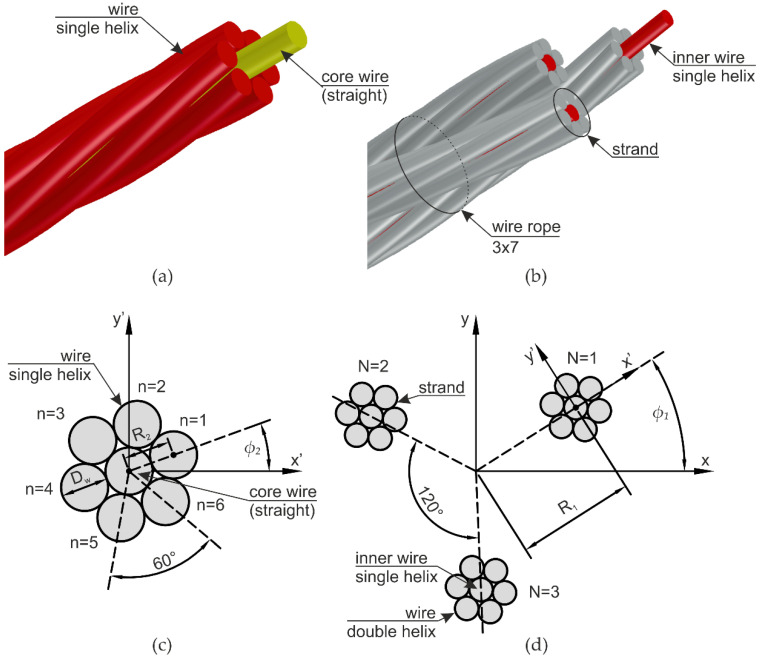
Geometry of the wire rope: (**a**) Simple 1 + 6 strand; (**b**) 3 × 7 wire rope structure; (**c**) single strand cross-section; (**d**) cross-section of a wire rope consisting of three strands.

One of the basic ropes is a straight strand (1 + 6), which was analyzed, for instance, in [1,2,21,24]. This simple strand consists of a straight center wire surrounded by six helical wires. An example of this strand is shown in Figure 4a. The straight core is marked in a yellow color, and the outer wires are depicted in red (red color in Figure 4 means that the wire geometry is described by circular helices). The analyzed 3 × 7 wire rope has a more complicated geometry and is made of three aforementioned strands spirally wrapped around itself, as can be seen in Figure 4b. Therefore, the centerline of the inner wire of each strand is defined through single helices (red color), in the same way as the outer wires of the 1 + 6 strand. Around each inner core wire lays a layer of six outer wires described by a double helix geometry marked in a gray color.

In the description of the wire rope geometry, it was assumed that in a plane perpendicular to the wire rope, the wire has a circular cross-section with the diameter *D_w_*. However, it has to be noted that the actual cross-section of the wire is elliptical in a plane normal to the rope, see, e.g., [3,13,24]. Herein, differences resulting from this simplification should be negligible and a similar assumption can be found in the paper [35]. In the first step, the geometry of a single strand was determined in a three-dimensional *x’y’z’* coordinate system, where the *z’*-axis coincides with the centroidal wire (Figure 4c). The analyzed wire rope consists of three such strands, so, in order to determine the final geometry, a new *xyz* coordinate system was assumed, in which the *z*-axis is in the longitudinal direction of the wire rope. In this coordinate system, the angle between each strand is 120 degrees. To determine the final coordinates, the points *x’*, *y’*, and *z’* have to be transformed from the *x’y’z’* systems of each strand into the *xyz* global system, as shown in the cross-section in Figure 4d. In this figure, for better clarity, the strands are separated from each other.

The final coordinates *x*, *y*, and *z* of the points of the helicoidal centerlines of the inner wire for each strand (*N* = 1, 2, 3), depending on the parameter *t*, are given by the following expressions:

(1) $$x_{N}\left(t\right)=R_{1}\cos\left(\beta_{N}\left(t\right)\right)$$

(2) $$y_{N}\left(t\right)=R_{1}\sin\left(\beta_{N}\left(t\right)\right)$$

(3) $$z_{N}\left(t\right)=t$$

The final coordinates *x*, *y*, and *z* of the points of the double-helical centerlines for each of the outer wires (*n* = 1, 2, 3, 4, 5, 6) of each strand (*N* = 1, 2, 3), depending on the parameter *t*, are defined as

(4) $$x_{N,n}\left(t\right)=\left(R_{2}\cos\left(\alpha_{n}\left(t\right)\right)+R_{1}\right)\cos\left(\beta_{N}\left(t\right)\right)-R_{2}\sin\left(\alpha_{n}\left(t\right)\right)\sin\left(\beta_{N}\left(t\right)\right)$$

(5) $$x_{N,n}\left(t\right)=\left(R_{2}\cos\left(\alpha_{n}\left(t\right)\right)+R_{1}\right)\cos\left(\beta_{N}\left(t\right)\right)-R_{2}\sin\left(\alpha_{n}\left(t\right)\right)\sin\left(\beta_{N}\left(t\right)\right)$$

(6) $$z_{N,n}\left(t\right)=t$$

The angles in the above formulas are defined by the following expressions:

(7) $$\alpha_{n}\left(t\right)=\frac{\pi }{3}\left(n-1\right)+\frac{2\pi }{P_{2}}t+\varphi_{2}$$

(8) $$\beta_{N}\left(t\right)=\frac{2\pi }{3}\left(N-1\right)+\frac{2\pi }{P_{1}}t+\varphi_{1}$$

In the above equations, *N* is the number of strands in the rope; *n* indicates the number of wires within the strand; *R*_1_ is the distance from the origin of the *xyz* global system to the center of the inner wire of the strand (initial radius of the helix of the inner wire of the strand); *R*_2_ is the distance from the center of the inner wire to the center of the outer wire in the strand (initial radius of the helix of the outside wire in the strand); *φ*_1_ denotes the initial rotation angle of the strand; *φ*_2_ stands for the initial rotation angle of the outer wire in the strand; and *P*_1_ and *P*_2_ are the pitch of the strand and the pitch of the single wire, respectively. The symbols are also shown in Figure 4c,d. The equations were used to construct the geometry of a straight section of the wire rope (see, e.g., the 3D model in Figure 1a).

The presented geometry description applies to a straight section of the 3 × 7 wire rope. In fact, the wire rope has an additional initial curvature, because of, e.g., its storage in rolls. The initial curvature is taken into account in experimental and numerical investigations.

## 3. Moment–Curvature Relationship

The moment–curvature relationship in beam theory is described in many books of mechanics of materials, e.g., [40,41]. Bernoulli’s assumption of flat sections is often taken into account since, in engineering applications, it is commonly fulfilled. Nevertheless, it cannot be applied to the analysis of a wire rope because the relative motion of wires and strands is possible and the strain field in a cross-section is not a smooth function.

It is assumed that some function of strains is defined over the cross-section domain $\varepsilon_{x}=\varepsilon_{x}\left(y,z\right)$ so that the integral of this function over the domain can be calculated. A strain is directly related to stress by Hooke’s law, which can be expressed mathematically as

(9) $$\varepsilon_{x}\left(y,z\right)=\frac{1}{E}\sigma_{x}\left(y,z\right)$$

where *E* is Young’s modulus.

From equations of statics it can be obtained that the moment resulting from the normal stresses *σ_x_* acting over the cross-section is equal to the bending moment *M*:

(10) $$\iint_{A}\sigma_{x}zdA=M$$

The substitution of expression (9) into (10) allows the bending moment *M* to be defined as a function of the material parameter and strain function:

(11) $$M=\iint_{A}\sigma_{x}zdA=E\iint_{A}\varepsilon_{x}zdA=\frac{E}{\rho }I$$

where $I=\rho \iint_{A}\varepsilon_{x}zdA$ and *ρ* is the beam’s curvature radius. *I* can be denoted as the moment of inertia of the cross-sectional area if Bernoulli’s assumption is fulfilled. In the current research, *I* denotes a certain constant expressed in mm^4^ determined experimentally.

Taking into account that the curvature *κ* is defined as the reciprocal of the radius of curvature *ρ*, Equation (11) can be rewritten as

(12) $$\kappa =\frac{1}{\rho }=\frac{M}{EI}$$

Under a constant bending moment, the deflection of the beam is described by the equation of the circle. The exact equation for the curvature of the beam, which expresses curvature through derivatives of the transverse displacement function *u* (the prime mark represents the derivative with respect to axial coordinate *x*), is

(13) $$\kappa =\frac{1}{\rho }=\frac{u^{\prime \prime }}{{\left[1+{\left(u^{\prime }\right)}^{2}\right]}^{\frac{3}{2}}}$$

## 4. Experimental Bending Testing

### 4.1. Test Specimens

Four-point bending tests were conducted for four 1.0 m long 3 × 7 wire ropes. The ropes were denoted as WR1, WR2, WR3, and WR4, respectively. Five bending tests were carried out for WR1, WR3, and WR4 wire ropes, and four tests for the WR2 rope, so the total number of tests was 19. As a consequence of this, the tests were denoted as follows: WRX-TY, where X refers to the wire rope number and Y indicates the test number.

The following specimens WR1, WR2, and WR4 were not prestretched before the bending test. The WR3 rope was initially prestretched to the force of 162 kN. The initial radius of curvatures prior to testing for WR1, WR2, WR3, and WR4 samples was 3693.5, 3145.0, 15,629, and 2723.3 mm, respectively. The large radius of curvature for the WR3 sample is due to the prestretching. In [20], in which a similar rope was tested, the initial radius of curvature was 1708.4 mm.

### 4.2. Test Stand

**Figure 5:**
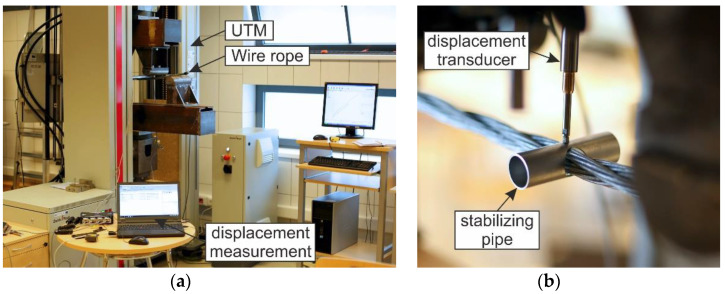
Test stand: (**a**) General view; (**b**) displacement transducer.

**Figure 6:**
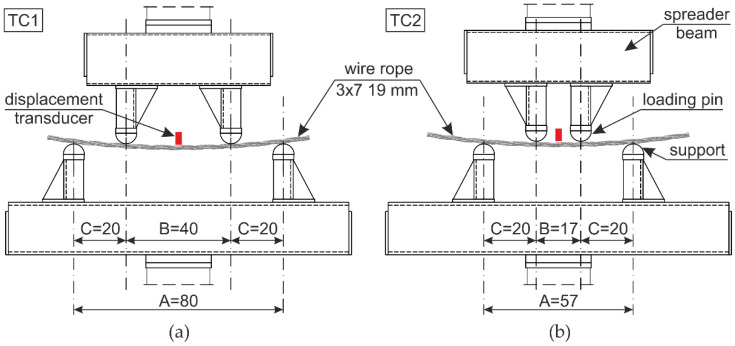
Test configurations: (**a**) TC1: *A* = 80 cm, *B* = 40 cm; (**b**) TC2: *A* = 57 cm, *B* = 17 cm.

The testing equipment and experimental setup for the 4-point bending of wire rope are shown in Figure 5 and Figure 6. The bending test was conducted using a 400-kN ZwickRoell Universal Testing Machine (UTM). In the conducted studies, two test stands were applied, as depicted in Figure 6. In the first test configuration (TC1), the distance between the supports equaled *A* = 80 cm and the distance between the two loading points was half of the distance between the supports and equal to *B* = 40 cm (Figure 6a). For the second configuration (TC2), the distances were as follows: *A* = 57 cm and *B* = 17 cm, respectively (Figure 6b). The tests were performed under constant deformation control with the speed of 20 mm/min. The diameters of the loading pins and the supporting pins were the same and equal to 80 mm.

**Figure 7:**
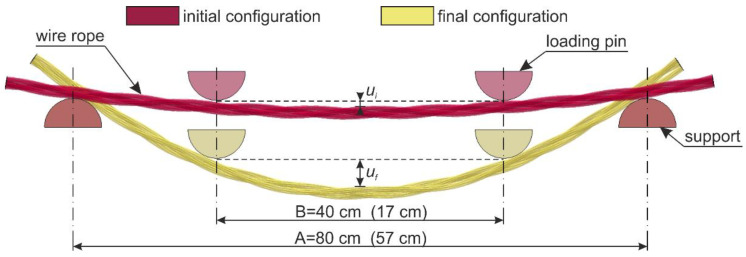
Measurement with a displacement transducer.

The wire ropes were equipped with a displacement transducer in the middle of the span. The inductive transducer was fixed to a spreader beam and moved with it during the test. In this way, the deflection of the rope in the middle of the span relative to the loading pins was measured. The method of the displacement measurement is shown in Figure 7. The displacements were calculated as *u_f_ − u_i_*. This measurement was used to determine wire rope curvatures, assuming that, between the loading points, the curvature was described by the equation of the circle. Because of the irregular geometry of the wire rope, the transducer had to be laid on the specimen via a stabilizing pipe, which prevented the transducer’s head from sliding on the rope (Figure 5b).

For the TC1 configuration, WR1, WR2, and WR3 ropes were tested, and for the TC2 configuration, WR4 rope was tested. It was decided that the three test samples would be used for tests in the first configuration to properly observe the initial bending-curvature dependence, in addition to one sample for the second configuration, so as to examine the bending response for larger rope curvatures. In order not to damage the transducer, the displacement of the UTM traverse was limited to 80 mm for WR1-WR3 ropes and 110 mm for WR4 rope.

### 4.3. Test Results

**Figure 8:**
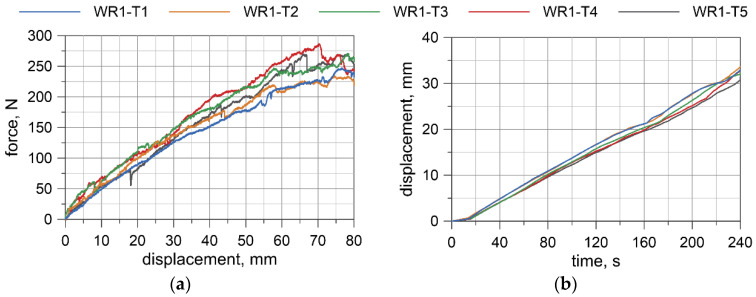
WR1: Load vs. displacement curve from a Universal Testing Machine (UTM) (**a**), and displacements from a transducer (**b**).

**Figure 9:**
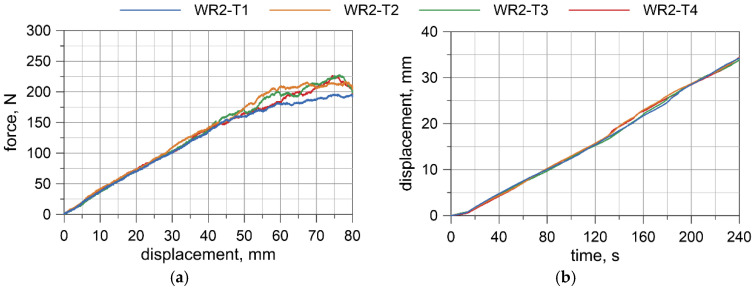
WR2: Load vs. displacement curve from a UTM (**a**), and displacements from a transducer (**b**).

**Figure 10:**
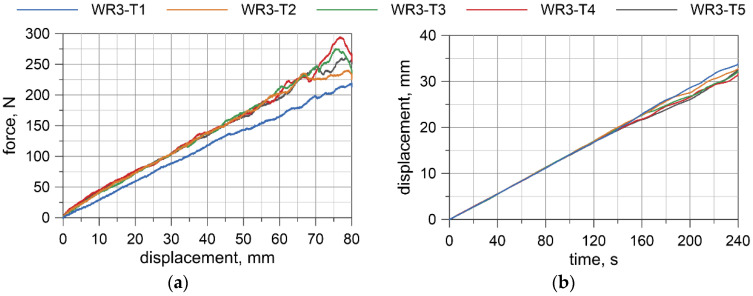
WR3: Load vs. displacement curve from a UTM (**a**), and displacements from a transducer (**b**).

**Figure 11:**
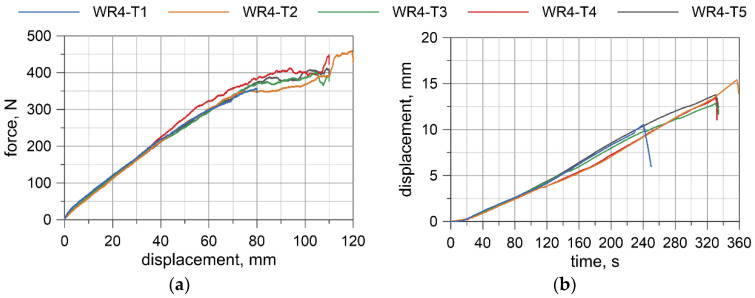
WR4: Load vs. displacement curve from a UTM (**a**), and displacements from a transducer (**b**).

Displacement-force curves were obtained from the testing machine. Deflections at the wire rope mid-span relative to the points where the load was applied were measured by the transducer. The results for all specimens are presented in Figure 8, Figure 9, Figure 10 and Figure 11. Considering the first TC1 configuration (WR1-WR3), the forces corresponding to the largest displacements are within the range of approximately 200–290 N. Wire rope deflections obtained from the displacement transducer are approximately 30–35 mm. 

For the TC2 configuration (WR4), the forces corresponding to the largest displacements are approximately 400–460 N and the displacements from the transducer do not exceed 16 mm. The first test WR4-T1 was conducted for the settings as in the TC1 tests, where the maximum displacement of the traverse was set to 80 mm. Due to the significant safety margin of the transducer, in the second test, WR4-T2, the traverse displacement was increased to 120 mm; however, for the last three tests (WR4-T3 to WR4-T5), it was decided to set the maximum displacement of the traverse to 110 mm.

Based on the acquired displacement curves, one can notice that there is a linear relationship for displacements at the mid-span. A linear relationship can also be observed between the force and the displacement measured by UTM, especially if considering the range up to 70% of the maximum displacement. The final radii of curvatures, after conducting all of the bending tests, for WR1, WR2, WR3, and WR4 wire ropes were 2862.9, 2575.5, 4047.8, and 2079.7 mm, respectively. The bending response of the prestretched WR3 rope is smoother than the response of the non-prestretched ropes, but the values of the maximum forces are comparable to the forces obtained in the non-prestretched WR1 rope test. The displacements measured at the midspan for WR1, WR2, and WR3 samples are similar. Because of the small differences between the non-prestretched and prestretched samples, it was decided to determine one final moment–curvature relationship applicable to both ropes.

## 5. Numerical Bending Testing

In this study, two test configurations were considered (TC1 and TC2, see Figure 6) and for each configuration, two numerical models were developed. In the first, the wire rope was modeled using beam FEs (B) and in the second, the rope was made of solid FEs (S). Therefore, the numerical tests are denoted as follows: TC1-B (configuration 1: *A* = 80 cm, *B* = 40 cm, the wire rope modeled with the use of beam FEs), TC1-S (configuration 1, the wire rope modelled using solid FEs), and TC2-B (configuration 2: *A* = 57 cm, *B* = 17 cm, beam FEs), TC2-S (configuration 2, solid FEs). The numerical simulations in which the wire rope was modeled using beam FEs will be abbreviated as “beam model” and those where solid FEs were used will be abbreviated as “solid model”, respectively.

The calculations were conducted using LS-DYNA software [42,43,44] (MPP double-precision R10.1.0) on the supercomputer Tryton, managed by the Academic Computer Centre (CI TASK) in Gdańsk, Poland. The keyword names are used in accordance with LS-DYNA documentation [42,43,44].

### 5.1. Numerical Model

**Figure 12:**
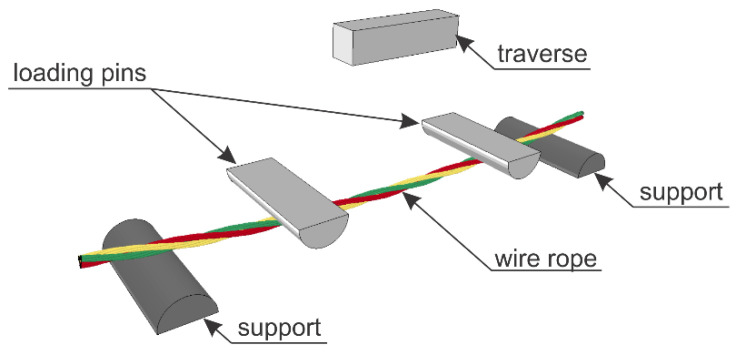
General view of the numerical test of 4-point bending.

The numerical model of 4-point bending consists of half-round supports and loading pins, the traverse, and the wire rope (Figure 12). Depending on the model, the following elements are varied: The spacing of the supports and loading points (depending on the configuration TC1 or TC2), and the wire rope model (beam or solid model).

Contact between the adjacent wires and between the wires and the UTM’s parts was modeled using a penalty-based mortar contact algorithm [42,45]. In the literature concerning simulations of wire ropes, one can find that values of the friction coefficient (FC) are mostly from 0.1 to 0.2 [10,11,16,17,21,22]. Considering the above, it was decided to assume that the friction coefficient was equal to 0.1. The impact of the friction coefficient value will be discussed in Section 5.2.2. The dead load was considered in the numerical simulations.

The nonlinear analyses were solved with the use of the full Newton solution method [42,44,46]. In the numerical testing of wire ropes, in the TC1 configuration, the simulation time was 240 s. For the TC2 configuration, the time was equal to 360 s. The time step for the simulations employing the beam wire rope model was 0.5 s, and for the calculations using the solid rope model, due to the size of the computational model, it was 1.0 s. It should be mentioned that, because these numerical analyses have a static character, the simulation time does not correspond to physical time and is simply a proportional load multiplier. Since the global displacement in simulations was considered to be large, the displacement norm for the convergence test (DNORM) was switched to 1 (increment vs. displacement over the current step) and the displacement convergence tolerance (DCTOL) was set to 0.001 for TC1-B, TC1-S, and TC2-S tests and 0.005 for the TC2-B test.

#### 5.1.1. Beam Model

**Figure 13:**
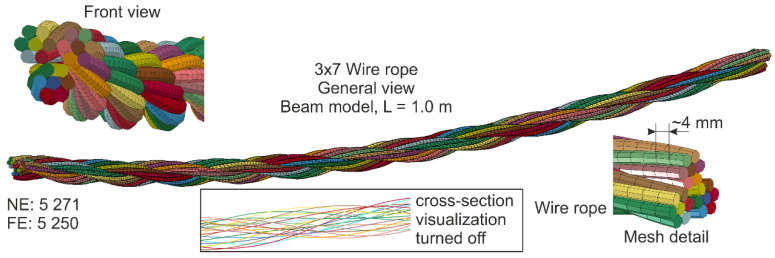
General view of wire rope made of beam finite elements (FEs) (visualization of the cross-section of the beam FEs is turned on).

The rope geometry was developed based on the equations presented in Section 3. Then, the model was bent to take into account the actual geometry of the ropes from experimental tests. The length of the wire rope equaled 1.0 m. Discretization of the single wire was conducted by means of Hughes–Liu beam-type elements (ELFORM = 1) [42,44] with the use of the 2 × 2 Gauss quadrature (four integration points in the cross-section). The length of a single beam element was approximately 4 mm and the diameter corresponded to the actual diameter of the wire and equaled 3 mm. The wires were modeled using the elastoplastic constitutive law with the material properties acquired from tensile testing carried out on samples cut from the wire rope. For the beam model, the number of nodes (NE) was 5271 and the number of FEs was 5250. The FEM beam model can be seen in Figure 13 (note that the visualization of cross-sections is displayed; additionally, in the box, the model showing the beam FEs without cross-section visualization is also presented).

#### 5.1.2. Solid Model

**Figure 14:**
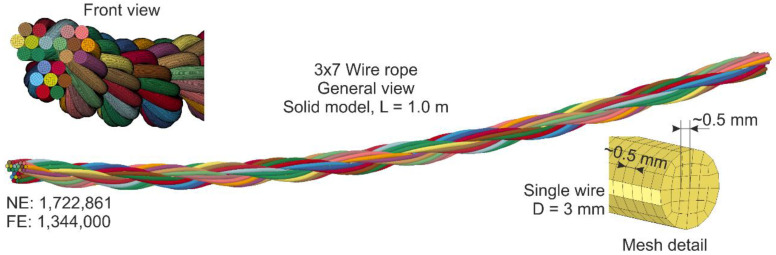
General view of wire rope made of solid FEs.

Similar to the beam model, the 3D geometry of the straight wire rope was constructed using the equations introduced in Section 3. Next, the model was bent to recreate the geometry of the wire ropes used in the experimental tests. The length of the wire rope was 1.0 m. The wires were modeled using 8 node constant stress solid elements (ELFORM = 1) [42,44] with a regular shape. Each wire had 32 solid FEs in the cross-section. The length of the side of the hexahedron elements was approximately 0.5 mm. The complete solid model of the wire rope is defined by NE = 1,722,861 and FE = 1,344,000. The view of the model and its details are shown in Figure 14. For better visibility, due to the fine mesh, the mesh is not displayed for the general view. The wires were assigned the same elastoplastic material model as for the beam wire rope model.

#### 5.1.3. Parts of the Universal Testing Machine

The numerical model of UTM includes half-round supports, half-round loading pins, and the traverse (Figure 12). The diameter of loading and supporting pins is 80 mm. All of the UTM parts were modeled using constant stress solid elements (ELFORM = 1) [42,44]. In the traverse, the length of the side of the solid elements equaled 10 mm, and for the support and loading pins, the side length varied from approximately 2.8 to 6.3 mm. The modeled UTM parts include 91,536 nodes and 81,460 solid FEs. The UTM was assigned the rigid material model [43] with properties corresponding to steel for contact analysis purposes. The loading pins were constrained to the traverse. The boundary conditions were defined through constraining all 6 degrees of freedom of bottom supports. For the remaining parts of the UTM (i.e., the loading pins and the traverse), vertical movement with a constant velocity was solely possible. The wire rope lay on the supports and it could freely slide on them during the loading, and the same was true in the physical tests.

### 5.2. Numerical Test Results

#### 5.2.1. Comparison with Experimental Results

**Figure 15:**
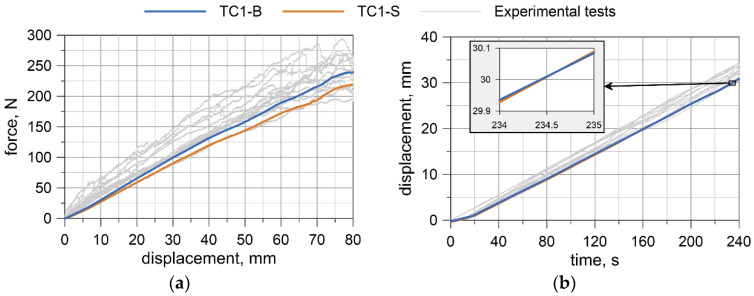
Comparison of numerical and experimental results for the TC1 configuration (*A* = 80 cm, *B* = 40 cm): Load vs. displacement of the traverse curve (**a**), and relative displacements at the midspan (**b**).

**Figure 16:**
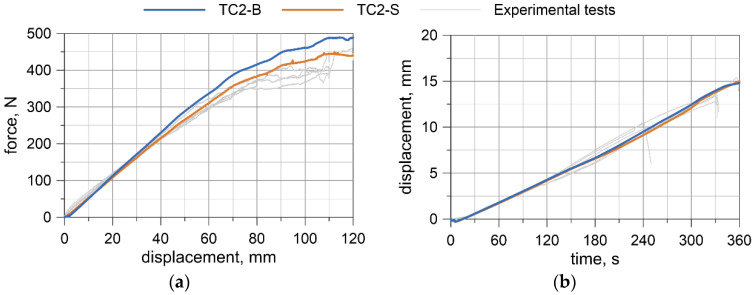
Comparison of numerical and experimental results for the TC2 configuration (*A* = 57 cm, *B* = 17 cm): Load vs. displacement of the traverse curve (**a**), and displacements from the transducer (**b**).

A comparison of the results from numerical simulations with the experimental ones is shown in Figure 15 and Figure 16. The experimental results are plotted in a gray color. The blue line represents the beam model (TC1/TC2-B), and orange represents the solid model (TC1/TC2-S). In Section 5.2.1 and Section 5.2.2, *T* denotes time and *D_T_* is the displacement of the traverse.

Considering the numerical results obtained from the TC1 configuration, the forces, as well as the displacements at the midspan, are approximately linear. The forces from simulations are in the lower range of those obtained from experiments, i.e., in most cases, for the same traverse displacement, the forces from experimental tests are greater than from numerical tests. The maximum force from the simulations for the beam and solid model is 239 and 219 N, respectively. The displacements of the rope at the midspan relative to the loading pins are also in the lower range of the results acquired experimentally. The right-hand side graph in Figure 15 portrays an additional window presenting comparison of displacements from *T* = 234 s to *T* = 235 s. The maximum displacement obtained from numerical calculations is almost 30.9 mm. For the TC1 configuration, the beam model reflects the experimental results a little more accurately.

By analyzing the second configuration, TC2, one can see that the force increases in a linear way to the value of the traverse displacement *D_T_* of approximately 70 mm; this is ~60% of the analyzed range (Figure 16a). Then, the slope of the curve changes. The maximum force obtained from the beam model is 489 N, whilst from the solid model, it is 440 N. The displacements at the midspan of the wire rope increase linearly, similar to the TC1 configuration, and coincide well with experimental outcomes (Figure 16b). The maximum displacement for the beam and solid model is 14.8 and 15.0 mm, respectively. For the TC2 configuration, the solid model shows better agreement with the experimental measurements, and the beam model appears to be slightly more rigid.

**Figure 17:**
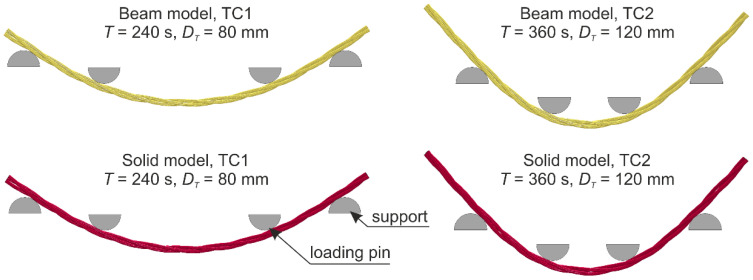
Wire rope deformation in numerical bending tests.

For the two considered configurations, the displacements in the middle of the wire rope span, relative to the loading pins, obtained from the beam model and the solid model, are similar. The final deformations for all models are depicted in Figure 17. The responses of the beam model are more rigid. For the TC1 configuration, the beam model delivered slightly better results, and for the TC2 configuration the opposite holds true. Notwithstanding, when considering the TC2 configuration in the range corresponding to the TC1 (i.e., the forces up to 250 N), the curves from the beam model and the solid model are similar, and, for the force range greater than 250 N, the solid model better reflects the real wire rope behavior.

#### 5.2.2. Analysis of Numerical Results

**Figure 18:**
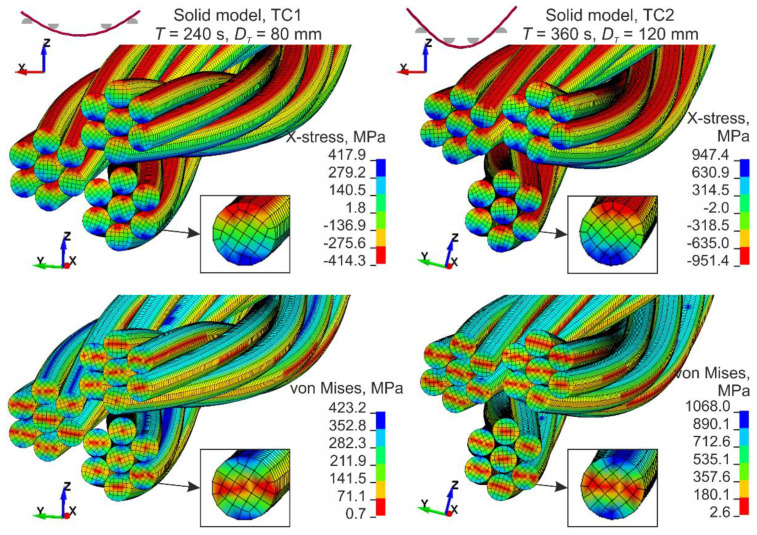
Solid model, stress distribution—cross-section at mid-span.

The stress distribution at the midspan from the solid model is shown on the top of Figure 18. The analysis of the stresses reveals that each wire works separately and under bending. The material of the bottom portion of each wire is stretched and the material of the top portion is compressed. The stress state of the single wire approximately corresponds to the state of pure bending. The stresses in the middle of the cross-section height are close to zero, which corresponds to the position of the neutral axis, and the stresses in the outmost fibers of the single wire have similar values, but opposite signs. By analyzing von Mises stresses (Figure 18, bottom), similar observations can also be made. In the middle part of the wires (red area in the figure), the stresses are significantly smaller than the stresses in the outmost fibers. Hence, the influence of the axial forces is negligible compared to the effects of the bending. In addition, stress analyses over the section between loading pins showed that the variability of the extreme stresses is approximately ±5%.

For maximum traverse displacement, in the TC2 configuration, the maximum stress is more than twice as high as the maximum stress of the TC1 configuration. The numerical simulation of the TC1 configuration shows that the stresses do not exceed the yield strength of 1118.5 MPa. However, for the TC2 configuration, the wire rope reached the plastic region. Maximum plastic deformations of 0.1‰ are located under loading pins. It should be highlighted that between the loading pins the wire rope remains in the elastic region. In Figure 18, by comparing the deformed cross-sections of TC1 and TC2 configurations, it can be seen that the strands move relative to each other during the bending and the displacement between the strands is greater for larger curvatures.

**Figure 19:**
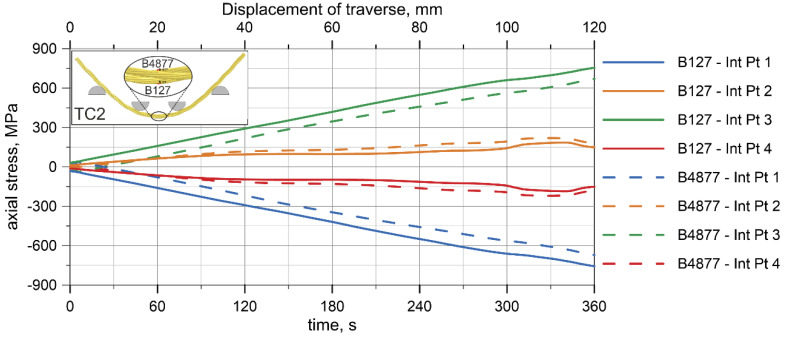
Beam model showing axial stresses at integration points.

Similar conclusions can be stated based on the results obtained from the beam wire rope model. Figure 19 shows the stresses at integration points (abbreviation: Int Pt) of the two beam FEs located near to the midspan of the wire rope for the TC2 configuration. For each FE, tensile stresses were obtained at two integration points and compressive stresses at the other two. These stresses have the same values, but opposite signs.

**Figure 20:**
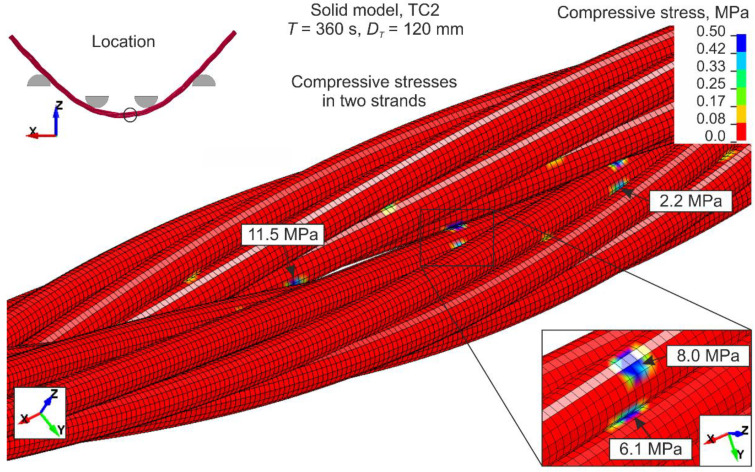
Contact compressive stress distribution. Note: Maximum of the fringe level was set to 0.5 MPa.

A contact stress analysis was conducted. The author’s preliminary studies revealed that the friction coefficient between the rope and UTM’s parts *FC_UTC_WR_* has more of an impact on the results than the friction coefficient between the wires *FC_WR_WR_*. These preliminary tests revealed that if the *FC_UTC_WR_* is reduced from 0.1 to 0.02 without changing *FC_WR_WR_*, the final force *F_UTC_* from the displacement-force curve can decrease by up to 6%. However, when only increasing *FC_WR_WR_* from 0.1 to 0.3, the *F_UTC_* force remains almost the same. If both coefficients, *FC_UTC_WR_* and *FC_WR_WR_*, are increased from 0.1 to 0.3, the *F_UTC_* force can increase by 20%. This indicates that the *FC_UTC_WR_* coefficient has a greater effect on the results than *FC_WR_WR_*. Finally, in the numerical models, one coefficient of friction equal to 0.1 was assumed (see Section 5.1). To thoroughly investigate the impact of friction, a detailed contact stress analysis was carried out. Figure 20 illustrates the normal contact stresses between the two strands in the neighborhood of the middle of the wire rope (see location in Figure 20) obtained from the solid model, with the TC2 configuration. Large stresses occur locally at points of contact between the wires. Higher values are observed between strands (e.g., 11.5 and 8.0 MPa) than between the wires within the single strand (e.g., 6.1 and 2.2 MPa). However, for most surfaces, the contact stresses equal zero. This results from the geometry of the wire rope; most surfaces are not in contact with other parts. A similar view of the contact stress distribution is shown in [17]. It is worth noting that the contact stresses between the wires are smaller than between the wires and UTM’s parts. For instance, the maximum contact compressive stress between the wire rope and loading pin is 65.4 and 153.9 MPa for TC1 (*T* = 240 s, *D_T_* = 80 mm) and TC2 (*T* = 360 s, *D_T_* = 120 mm) configurations, respectively. This also confirms that the friction coefficient between the wires and the UTM affects the results more than the friction coefficient between the wires.

Numerical simulations correctly reflected the wire rope performance during experimental tests, giving a reliable insight into the wire rope behavior. Despite minor differences, it can be stated that both models provide comparable bending responses; therefore, the developed models can be considered as validated.

## 6. Results and Discussion

### 6.1. Moment–Curvature Relationship for the Wire Rope

The bending moment was determined based on the forces acquired from UTM (Figure 8a, Figure 9a, Figure 10a, Figure 11a), and the curvature of the wire rope was calculated based on the measurements recorded by the displacement transducer (Figure 8b, Figure 9b, Figure 10b, Figure 11b).

The first step was to determine the bending moment. Because a symmetrically loaded simple beam was considered, it was assumed that the central region of the wire rope, between the loading pins, underwent pure bending. The bending moment *M* was calculated from the following relation:

(14) $$M=\frac{FC}{2},$$

where *F* is the force from UTM and *C* denotes the distance from the loading pin to the support (see. Figure 6).

**Figure 21:**
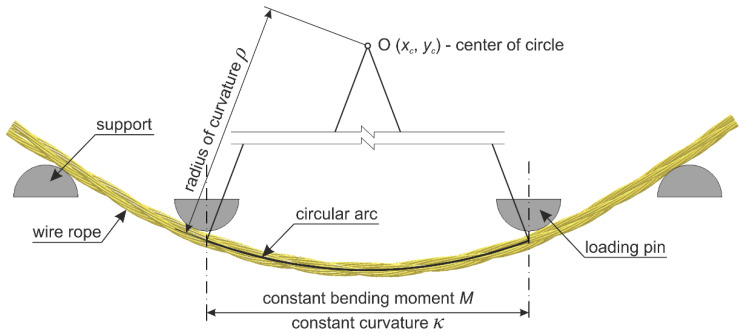
Determination of the radius of curvature for the deformed wire rope.

The second step was to determine the curvature of the bent wire rope. Between the loading pins, the curvature was constant and the deflection curve was described by the equation of the circle (Figure 21):

(15) $${\left(x-x_{c}\right)}^{2}+{\left(y-y_{c}\right)}^{2}=\rho^{2},$$

where *x_c_* and *y_c_* are the center coordinates and *ρ* is the radius. Since the coordinates of three points (two loading pins and the displacement at the mid-span) were known, the equation of the circle could be determined.

**Figure 22:**
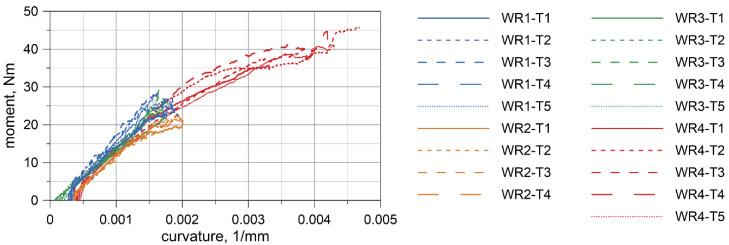
Moment–curvature relationships obtained from experimental tests.

For each laboratory test, the bending moment *M* acting on the wire rope was determined from Equation (14). When determining the radius *ρ* from Equation (15), it was taken into account that the arc of the circle coincided with the longitudinal axis of the curved rope (by adding half of the rope thickness, see Figure 21) and that the wire rope had been initially bent before the tests (see *u_i_* deflection in Figure 7). The sought curvature *κ* is the reciprocal of the radius *ρ*. In this way, the moment–curvature relationships were determined for all experimental tests, as shown in Figure 22.

**Figure 23:**
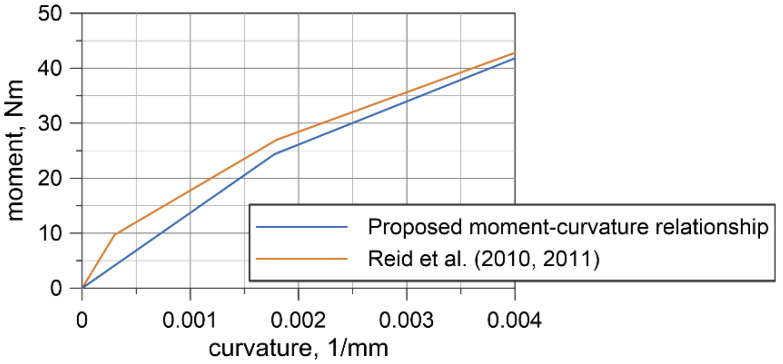
Moment–curvature relationship for 3 × 7 19-mm wire rope.

To determine the final moment–curvature relationship, the curve was fitted to the set of the experimental discrete points utilizing the method of least squares. It was assumed that this final curve must be concave. The determined nonlinear elastic relationship is shown in Figure 23, in which it is also compared in the analyzed range of curvatures to the curve proposed by Reid et al. [20].

The curvature and the bending moment are related by Equation (12). Based on the determined relationship, the flexural stiffness *EI* at point (0,0) for the wire rope is 13.7 × 10^6^ Nmm^2^. Assuming that the modulus of elasticity of the non-prestretched wire rope is 79.9 GPa [35], the moment of inertia of the cross-sectional area of the wire rope *I* is equal to 172 mm^4^.

### 6.2. Discussion

The four-point bending tests of the 3 × 7 19-mm wire rope indicate that in the initial range of curvatures, the wire rope behaves linearly. The bending response of the prestretched wire rope is smoother than the response of non- prestretched rope. However, the force values are similar for non-prestretched, as well as for prestretched, rope (compare Figure 8a and Figure 10a). Moreover, the displacements in the middle of the span are similar (see, e.g., Figure 8b and Figure 10b). Therefore, it was decided not to independently determine the bending characteristics for prestretched and non-prestretched ropes, expecting that the discrepancies would be insignificant. With the potential use of this curve to analyze cable barrier systems in numerical simulations, this approach can allow considerable facilitation. Furthermore, as mentioned in Section 2.1, the properties of the rope can alter over time under real road and environmental conditions, so this approach seems even more reasonable.

Based on the experimental tests, the final moment–curvature relationship in the range of curvatures up to 0.004 mm^−1^ was determined (Figure 23). The obtained curvature corresponds to bending the wire rope into a circle with a radius of 25 cm. This relationship is comparable to that proposed by Reid et al. [20,35]; however, it should be noted that Reid’s relationship covers a higher range of curvatures, up to 0.05 mm^−1^. The curves, compared in Figure 23, differ in the initial range of curvatures. The proposed curve is characterized by a smaller slope at the origin point and consequently, the obtained flexural rigidity of 13.7 × 10^6^ Nmm^2^ is lower than the value of 32.3 × 10^6^ Nmm^2^ given in [35]. Considering the cross-section of the wire rope as either 21 independent wires or as a single solid cross-section, the moment of inertia is 83.5 and 2660 mm^4^, respectively. The experimentally determined moment of inertia *I* = 172 mm^4^ indicates that the wire rope works more as a set of 21 separated wires than as one solid section. A similar observation can be found in [35]. The reason for this is that the wrap of the rope, contact surfaces, and friction forces between the wires do not provide sufficient cross-sectional bonding to allow it to work as a single solid structure.

Detailed stress analysis demonstrates that each wire is under bending and that the influence of the axial forces is insignificant compared to the effects of the bending. In the analyzed wire rope section, between the loading points, the strains of the wires are in the elastic range. However, the strains of the wires placed directly under the loading pins are locally in the plastic range. This fact may suggest that even if the striking of the vehicle into the wire ropes does not exert substantial forces on the ropes, and plastic strains may occur locally in the place of contact between the rope and the vehicle.

The contact stresses occur locally between the wires and this results from the architecture of the wire rope. The values of contact pressure between the wires are small compared to the contact stresses between the rope and the UTM’s parts. This indicates that the friction coefficient between the adjacent wires influences the test result a little. It is due to the fact that the ratio between the sum of contact surfaces and the sum of outside surfaces of the wires is small and the relative displacements between wires are negligible. A similar conclusion can be found in the papers [12,24].

The rope modeled using beam FEs gives a more rigid bending response than the model utilizing solid FEs. However, the differences are small and the overall behavior of the two considered models is similar. It should be emphasized that the number of FEs of the beam rope model accounts for 0.4% of the number of FEs used in the solid rope model. This directly influences the calculation time and the necessary memory and disc resources. The outcomes of the numerical simulations are similar to the experimental ones; consequently, the tests can be considered reliable.

## 7. Conclusions

Wire rope is a complicated structure that is commonly used in many fields of civil and mechanical engineering. Previously, mainly analytical analyses were carried out. Nowadays, numerical simulations are often employed in wire rope investigations. This paper presents research on the bending properties of 3 × 7 19-mm wire rope used in road safety equipment. The most important achievements include the following:

- Developing equations for a wire rope geometry;
- Developing two advanced nonlinear FE models of wire rope utilizing beam and solid finite elements;
- Conducting an analysis of 19 experimental tests and four numerical simulations of four-point bending. The simulations were validated against the experimental results;
- Detailed analysis of numerical results including both cross-sectional and contact stress analyses;
- Determination of the nonlinear elastic moment–curvature relation for the wire rope.

The aforementioned actions enabled an in-depth analysis of the wire rope work mechanism under bending conditions and the formulation of conclusions. The most important discoveries and findings are listed below:

- The responses of non-prestretched and prestretched wire rope in the range of curvatures up to 0.004 mm^−1^ (i.e., the radius of curvature equals 25 cm) are similar and one moment–curvature relation is assumed for both prestretched and non-prestretched ropes;
- In the analyzed range of curvatures, the wire rope worked in the elastic range. Plastic strains in wires appeared solely locally under the loading pins. This suggests that in real-life accidents, the wire rope may work in the elastic range as well; however locally, in the vicinity of a point where a vehicle impacts the barrier, plastic strain may emerge;
- The considered wire rope works more as a set of 21 separated wires than as one single solid section;
- The interwire friction coefficient does not substantially affect the results. This is due to the wire rope geometry as most of the wire surfaces are not in contact with other wires.
